# Microwave-Hydrothermal Synthesis of SnO_2_-CNTs Hybrid Nanocomposites with Visible Light Photocatalytic Activity

**DOI:** 10.3390/nano7030054

**Published:** 2017-03-03

**Authors:** Shuisheng Wu, Weili Dai

**Affiliations:** 1 College of Chemistry and Chemical Engineering, Hunan Institute of Engineering, Xiangtan 411104, China; 2 Key Laboratory of Jiangxi Province for Persistent Pollutants Control and Resource Recycle, Nanchang Hangkong University, Nanchang 330063, China; dwl_1981@yeah.net

**Keywords:** SnO_2_, carbon nanotubes, nanocomposite, photocatalytic

## Abstract

SnO_2_ nanoparticles coated on carbon nanotubes (CNTs) were prepared via a simple microwave-hydrothermal route. The as-obtained SnO_2_-CNTs composites were characterized using X-ray powder diffraction, Raman spectroscopy, and transmission electron microscopy. The photocatalytic activity of as-prepared SnO_2_-CNTs for degradation of Rhodamine B under visible light irradiation was investigated. The results show that SnO_2_-CNTs nanocomposites have a higher photocatalytic activity than pure SnO_2_ due to the rapid transferring of electrons and the effective separation of holes and electrons on SnO_2_-CNTs.

## 1. Introduction

In recent years, a great deal of effort has been devoted to decomposing harmful organic pollution [1,2]. Compared with the conventional oxidation processes, semiconductor photocatalysis is an attractive candidate because of its many advantages, such as complete mineralization of the pollutants, application of the ultraviolet (UV) or solar light, and low cost [3,4,5,6]. Considering that sunlight contains only 5% ultraviolet light (λ < 380 nm), there is a need for the development of visible light-responsive photocatalysts with high activity.

SnO_2_—a stable and large n-type bandgap (E_g_ = 3.6 eV) semiconductor [7,8]—has excellent photoelectronic properties, gas sensitivity, and superior chemical stability, which has already been used in sensors [9], solar cells [10], lithium-ion batteries [11], and photocatalysts [12]. Recently, a nanocomposite of SnO_2_-CNTs (carbon nanotubes) and SnO_2_ coated on nitrogen-doped carbon nanotubes was synthesized, and exhibited excellent photocatalytic activity due to the electron transfer between SnO_2_ and CNTs [13,14]. However, the photocatalytic properties of SnO_2_-CNTs prepared by microwave-hydrothermal method have been scarcely investigated.

CNTs-metal oxide hybrid materials have been suggested as a new material for heterogeneous photocatalysis due to the large surface area and unique electrical properties of CNTs. Some studies on TiO_2_-CNTs composites have proved that the conductive structure of the CNTs facilitates accepting and transferring the light-excited electrons from the conduction band (CB) of the semiconductor to the CNTs surface, which hampered the recombination of the electron-hole pairs [15,16,17,18]. In addition, CNTs as supports would probably promote the catalysts separation during the recycling use compared with solo semiconductor nanoparticles [19].

Microwave-hydrothermal reaction has been used as an effective method for the synthesis of semiconductor nanoparticles. The microwave hydrothermal process used only 0.5–2 h for synthesis of rutile titania, while conventional hydrothermal process needed more than 72 h for rutile phase using the same chemicals. They controlled particle size, morphology, and polymorph of titania under microwave-hydrothermal conditions by adjusting the various reaction parameters, such as pH, heating time, and pressure [20]. Very recently, Ponzoni et al. successfully synthesized lanthanum-doped bismuth ferrites using a microwave-assisted hydrothermal method, which strongly confirms the effectiveness of microwave hydrothermal reaction as a fast method for the synthesis of nanoparticles having specific properties [21]. Yin et al. synthesized nitrogen-doped titania nanoparticles by the microwave hydrothermal method in 5–60 min, and they showed excellent photocatalytic ability for the oxidative destruction of nitrogen monoxide under irradiation by both visible light and UV light [22,23]. In this work, SnO_2_-CNTs hybrid nanostructures were prepared via simple microwave-hydrothermal method and the photodegradation of Rhodamine B (RhB) was investigated for the first time. In addition, the photocatalytic mechanism of SnO_2_-CNTs is also discussed in detail.

## 2. Materials and Methods

Synthesis of SnO_2_-CNTs: The synthesis of SnO_2_-CNTs was carried out via microwave- hydrothermal method optimized by Cao research group for pure SnO_2_ [12]. In a typical procedure, 2 mmol of SnCl_4_·5H_2_O and 30 mg CNTs were added to a stirred deionized water (20 mL) while stirring for 10 min at room temperature. Then, 20 mL Lysin solution (10 mmol) was added dropwise to above solution. After being vigorously stirred for 30 min at room temperature, the final clear solution was transferred to a Teflon vessel of the MDS-6 (Microwave Digestion/Extraction System, Shanghai Sineo Microwave Chemical Technology Co. Ltd., Shanghai, China). The reaction mixture was heated up to 180 °C in 3 min, then this temperature was maintained for 10 min. After the Teflon vessel was cooled down, the as-prepared powders were repeatedly washed with the distilled water and ethanol several times, filtered, and dried in an oven at 60 °C.

Characterization of SnO_2_-CNTs: Samples were characterized by using X-ray diffraction (XRD) with a Bruker D8 Advance diffractometer (Karlsruhe, Germany) using Cu Kα (λ = 1.5418 Å) and operating at 40 kV and 40 mA. Transmission electron microscopy (TEM) images were obtained by using a JEM-2100 transmission electron microscope (Japan Electron Optics Laboratory Co. Ltd., Tokyo, Japan) operating with an accelerating voltage of 100 kV. Raman spectrum was recorded on a RM-1000 (Renishaw plc, New Mills, UK) with excitation from the 514 nm line of an Ar-ion laser with a power of about 5 mW. Infrared spectra (IR) measurements were carried out on a NICOLET 560 (Thermo Nicolet Corporation, Madison, WI, USA) Fourier transform infrared spectrophotometer.

Photocatalytic activity test of SnO_2_-CNTs: The photocatalytic activities of the as-synthesized SnO_2_-CNTs were evaluated in terms of the degradation of RhB in an aqueous solution. A 500-W Xenon lamp (Institute of Electric Light Source, Beijing, China) with a maximum emission of about 470 nm was the visible light source. A cutoff filter (λ > 420 nm) controlled the light’s wavelength. Sample (50 mg) was suspended in 50 mL of an aqueous solution of 10^−5^ mol/L RhB. The solution was continuously stirred for about 30 min at room temperature to ensure the establishment of an adsorption–desorption equilibrium among the photocatalyst, RhB, and water before irradiation with visible light. The concentration of RhB was monitored by using a UV-1600 UV-Vis spectrometer (Shanghai Meipuda Instrument Co. Ltd., Shanghai, China).

## 3. Results and Discussion

**Figure 1:**
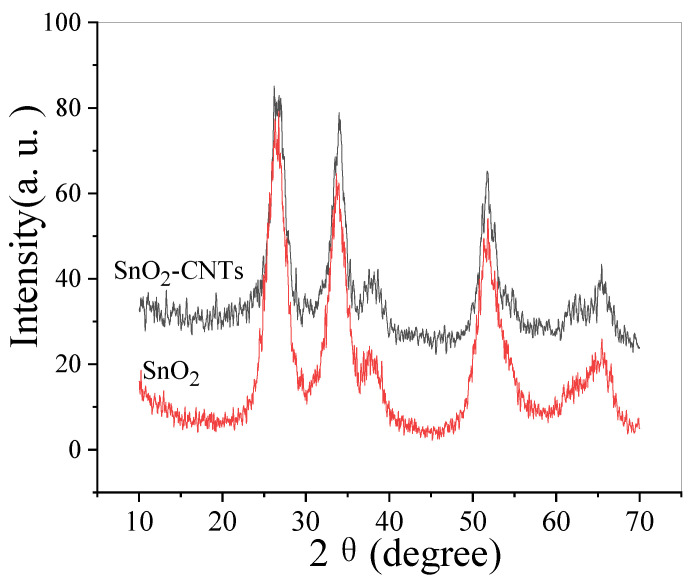
XRD patterns of SnO_2_-CNTs and SnO_2_.

To characterize the crystalline structure of the samples, the XRD patterns of SnO_2_ and SnO_2_-CNTs nanocomposites are displayed in Figure 1. For SnO_2_ and SnO_2_-CNTs nanocomposite catalysts, all of the diffraction peaks observed in the XRD patterns belong to the tetragonal rutile structure of SnO_2_ (JCPDS card No. 41-1445). The diffraction angle for SnO_2_-CNTs composites at 2θ = 26.3°, 33.6°, and 51.8° can be assigned to the 110, 101, and 211 planes of the cassiterite SnO_2_, respectively. It is noteworthy that the characteristic peaks of the CNTs can hardly be identified from SnO_2_-CNTs nanocomposite. The reason may be that the main peak of CNTs at 25.9° is overlapped with the main peak of anatase SnO_2_ at 26.6°. The SnO_2_ average crystalline size can be estimated by Scherrer’s formula:

*D* = *K*λ/*B*cosθ (1)

where *D* is the grain diameter, *K* (0.89) is the shape factor, λ is the X-ray wavelength of Cu Kα radiation (0.154 nm), θ is the Bragg angle, and β is the experimental full-width half-maximum (FWHM) of the respective diffraction peak. The crystallite grain size of SnO_2_ were calculated to be about 4.5 nm for pure SnO_2_ and 5.0 nm for SnO_2_-CNTs nanocomposite.

**Figure 2:**
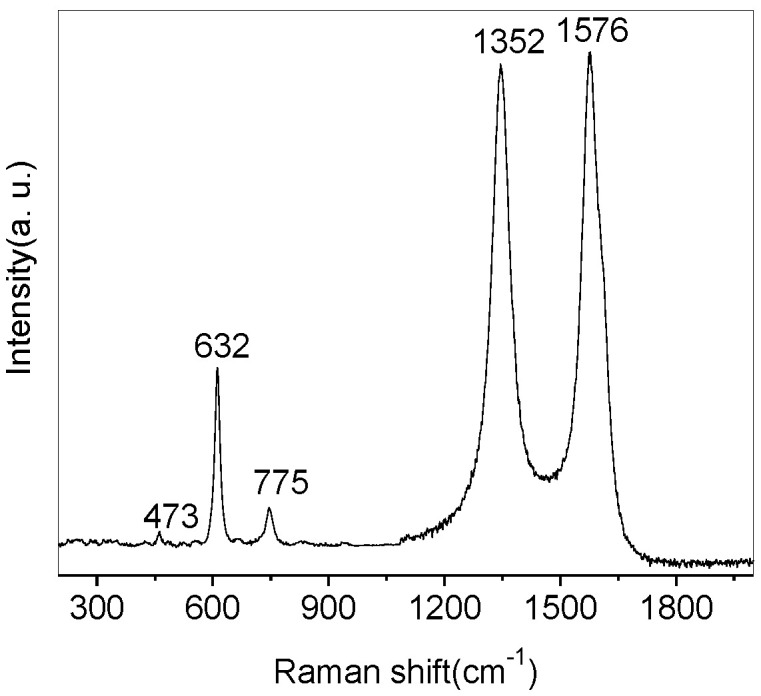
Raman spectrum of SnO_2_–CNTs nanocomposites.

Figure 2 shows the Raman spectrum SnO_2_-CNTs nanocomposites. Typically, the Raman spectrum exhibits bands at 473, 632, 775, 1354, and 1596 cm^−1^ in the range of 200–2000 cm^−1^ The Raman peaks appearing at 473 cm^−1^ can be attributed to the E_g_ mode, 632 cm^−1^ to the A_1g_ mode, and 775 cm^−1^ to the B_2g_ mode of SnO_2_, respectively [24]. The Raman peaks appearing at 1576 cm^−1^ and 1352 cm^−1^ can be attributed to the G band corresponding to the *sp*^2^ hybridized carbon and the *D* band originating from the disordered carbon of CNTs. The Raman analysis demonstrates that the as-synthesized samples comprised rutile-type SnO_2_ and CNTs.

**Figure 3:**
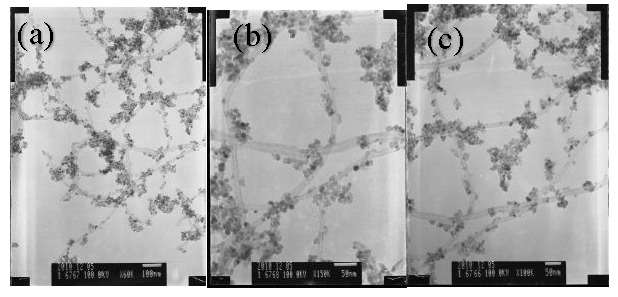
Transmission electron micrographs (TEM) of SnO_2_-CNTs nanocomposites. (**a**) low magnification; (**b**,**c**) high magnification

Figure 3 shows the TEM images of SnO_2_-CNTs nanocomposites. It can be found that the carbon nanotubes with an external diameter of 20–30 nm are uniformly distributed. After coating with SnO_2_, all CNTs are uniformly coated with a layer of SnO_2_ nanoparticles, and very few free nanoparticles were found. The SnO_2_ nanoparticles deposited on the surface of CNTs are separated, and the particle size is about 5–8 nm (in agreement with the XRD data), estimated using the Scherrer equation.

**Figure 4:**
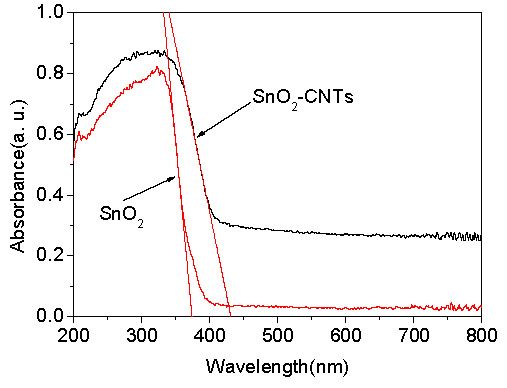
Ultraviolet-visible diffuse reflection (UV-Vis DRS) spectra of SnO_2_-CNTs and SnO_2_.

The ultraviolet-visible diffuse reflection (UV-Vis DRS) spectrum of SnO_2_ and SnO_2_-CNTs were measured using a UV-Vis spectrophotometer with an integrating sphere (Hitachi U-3900), as shown in Figure 4. The absorption threshold of pure SnO_2_ is 372 nm. It presents a strong absorption band only in the UV region. However, SnO_2_-CNTs sample extended the absorption range to the visible region, and the absorption edge red-shifted from 372 nm to 425 nm compared to pure SnO_2_, suggesting that SnO_2_-CNTs has the potential to be an efficient visible-light-activated photocatalyst.

**Figure 5:**
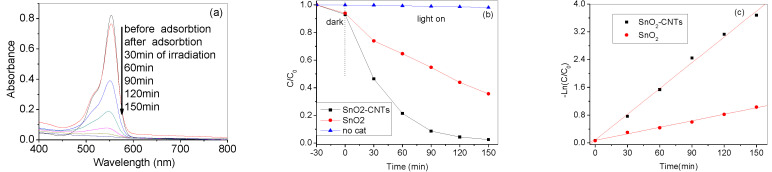
(**a**) The UV-Vis spectrum changes during Rhodamine B (RhB) (10^−5^ M) photodegradation by SnO_2_-CNTs photocatalysts; (**b**) Plots of photocatalytic degradation of RhB concentration vs. irradiation time in the presence of SnO_2_-CNTs samples; (**c**) dependence of −ln(*C*/*C*_0_) on irradiation time.

The photocatalytic activities of the as-synthesized nanocomposites were investigated by the photocatalytic oxidation of RhB dye. Figure 5a shows that the spectrum changes during RhB (10^−5^ mol/L) photodegradation by SnO_2_-CNTs with different reaction time. The normalized temporal concentration changes (*C*/*C*_0_) of RhB during the photocatalytic process are proportional to the normalized maximum absorbance (*A*/*A*_0_), as can be derived from the change in the RhB absorption profile at a given time interval. The blank experiments show that the degradation of RhB is less than 5% only under visible light irradiation at 150 min (Figure 5b). With the increase of irradiation time, the intensity of the maximum adsorption peak located near 553 nm gradually decreased, indicating the degradation of the RhB dye solutions. When SnO_2_-CNTs nanocomposites were used as photocatalysts, the degradation of RhB reached 95.60% after 150 min, which is much higher than pure SnO_2_, with 64.4% degradation of RhB.

To further understand the reaction kinetics of RhB degradation, the apparent pseudo-first-order model expressed by Equation (2) was applied in our experiments [25]:

−ln(*C*/*C*_0_) = *kt* (2)

where *k* is the apparent pseudo-first-order rate constant (min^−1^), *C* is the RhB concentration in aqueous solution at time *t* (mol/L), *t* is irradiation time and *C*_0_ is initial RhB concentration (mol/L). Figure 5c shows the first-order linear fit from the experimental data; the *k* value of SnO_2_-CNTs and SnO_2_ were 0.07434 (*R*^2^ = 0.99) and 0.01197 (*R*^2^ = 0.99) min^−1^. The result shows that the SnO_2_-CNTs nanocomposite is a much more effective photocatalyst than pure SnO_2_.

**Figure 6:**
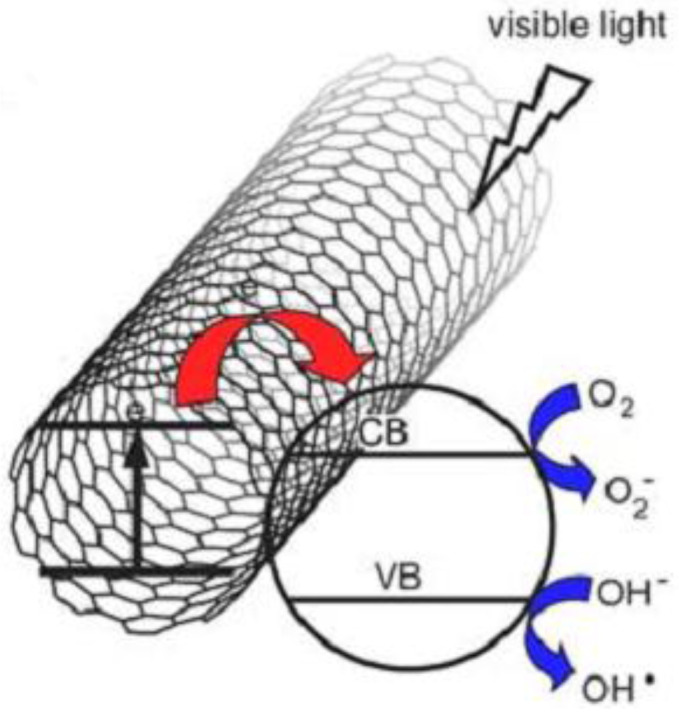
Proposed photocatalytic mechanism of SnO_2_-CNTs nanostructures. VB: Valence band; CB: conduction band.

The photocatalytic performance of SnO_2_-CNTs is superior to that of SnO_2_ due to the synergy effect of CNTs as a photosensitizer. The most probable mechanism for the photocatalytic degradation of RhB dye by SnO_2_-CNTs is proposed (Figure 6). Under visible irradiation, considering the semiconducting property of carbon nanotubes, CNTs may absorb visible light and inject the photo-induced electron into the SnO_2_ conduction band, which can trigger the formation of very reactive radicals, superoxide radical ion O_2_·^−^, and hydroxyl radical OH·—both responsible for the degradation of the organic compound. The probable electron transfer mechanism between carbon and semiconductor was experimentally supported by the observed enhanced photocurrent of the composite materials in other investigations [26,27,28,29]. The rapid transferring of electrons on SnO_2_-CNTs and the larger production of reactive radical species coming from oxygen reduction probably result in the enhanced photocatalytic activity of SnO_2_-CNTs hybrid nanostructures for the degradation of RhB.

## 4. Conclusions

SnO_2_ nanoparticles were successfully coated on CNTs via a simple microwave-hydrothermal method. The composites showed excellent photocatalytic activity compared to pure SnO_2_. The rapid transferring of electrons on SnO_2_-CNTs led to the dramatically enhanced photoactivity. The SnO_2_-CNTs composites would be an excellent photocatalyst for application in environmental protection.
