# Correction: Wu, S.; Dai, W. Microwave-Hydrothermal Synthesis of SnO_2_-CNTs Hybrid Nanocomposites with Visible Light Photocatalytic Activity. *Nanomaterials* 2017, *7*, 54

**DOI:** 10.3390/nano15231776

**Published:** 2025-11-26

**Authors:** Shuisheng Wu, Weili Dai

**Affiliations:** 1 College of Chemistry and Chemical Engineering, Hunan Institute of Engineering, Xiangtan 411104, China; 2 Key Laboratory of Jiangxi Province for Persistent Pollutants Control and Resource Recycle, Nanchang Hangkong University, Nanchang 330063, China; dwl_1981@yeah.ne

In the original publication [1], there was a mistake in the XRD patterns of the SnO_2_-CNTs and SnO_2_ in Figure 1, as published. Upon careful examination, the original data was found to be incorrect when plotted using Origin software. The corrected Figure 1 appears below. The authors state that the scientific conclusions are unaffected. This correction was approved by the Academic Editor. The original publication has also been updated.

## Figures and Tables

**Figure 1:**
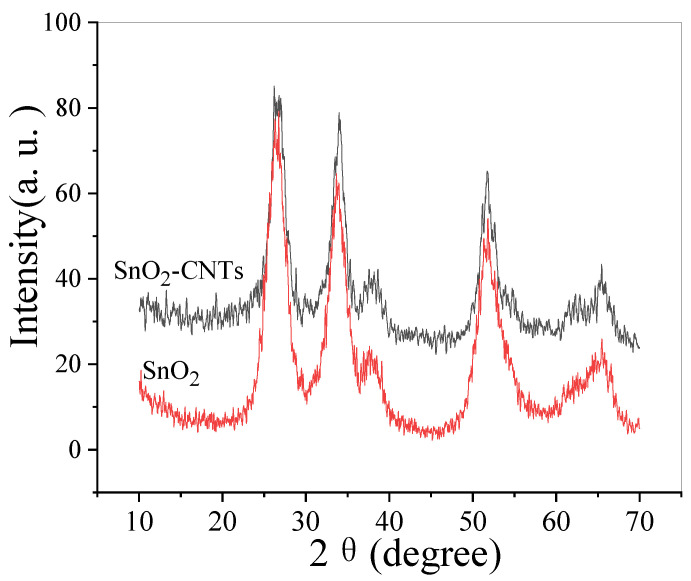
XRD patterns of SnO_2_-CNTs and SnO_2_.
